# Double burden of malnutrition among children under 5 in poor areas of China

**DOI:** 10.1371/journal.pone.0204142

**Published:** 2018-09-17

**Authors:** Yan Zhang, Xiaona Huang, Yuning Yang, Xiaoli Liu, Chenlu Yang, Anqi Wang, Yan Wang, Hong Zhou

**Affiliations:** 1 Department of Women’s Health, Beijing Obstetrics and Gynecology Hospital, Capital Medical University, Beijing Maternal and Child Health Care Hospital, Beijing, China; 2 Department of Maternal and Child Health, School of Public Health, Peking University, Beijing, China; 3 United Nations International Children’s Emergency Fund China, Beijing, China; 4 Department of Epidemiology, Bloomberg School of Public Health, Johns Hopkins University, Baltimore, United States of America; University of Kwazulu-Natal, SOUTH AFRICA

## Abstract

**Objective:**

The aim of this study was to investigate the nutritional status and its risk factors among children under 5 years, with special focus on the coexistence of under and over nutrition in population level.

**Methods:**

We enrolled 6,570 children under 5 years among 26 counties in poor areas of China. Prevalences of malnutrition (stunting, underweight, wasting, overweight) were calculated. Overweight was evaluated using two indicators–weight for height Z score (WHZ) and body mass index for age Z score (BAZ), and results were compared.

**Results:**

The total prevalence of malnutrition was 19.2%. The prevalence of stunting and overweight were as high as 8.4% and 8.8%, respectively. The BAZ-estimated prevalence of overweight was 9.9%, which was higher than the WHZ-estimated prevalence (p<0.001). Children older than 12 months age, within a minority group, with a caregiver of illiteracy/primary education level were significantly associated with stunting in multilevel regression models (p<0.05). Children younger than 24 months age and boys were significantly associated with overweight (p<0.05).

**Conclusions:**

Stunting and overweight were coexisted in surveyed areas. In conclusion, BAZ tended to overestimate the overweight prevalence compared with WHZ. While with the raising problem of childhood overweight, stunting should still be on the agenda for the poor areas in China. To improve children’s nutritional status in poor areas of China, caregivers with children at high risk of malnutrition should be guided for healthy feeding practices.

## Introduction

In China, the child undernutrition issue has been regarded as one of the most severe public health problems in poor areas, which are mainly located in western and middle rural China. During the past decades, the nutritional status of children under 5 in the overall China has improved dramatically, however, gaps still existed between poor areas and other areas [1–4]. For example, in 2013, the prevalence of stunting, underweight and wasting among children under 5 in poor areas were reported 18.7%, 5.2% and 3.0%, respectively, in contrast to the prevalences of 8.1%, 2.4%, and 1.9% in the overall China[3].

Child malnutrition includes not only undernutrition, but overnutrition, i.e., overweight and obesity[5]. Few studies have recognized the problem of overnutrition in poor areas in China. Piernas et al. reported the prevalence of child overweight and obesity was 13.5% and 9.9% respectively among a small sample of children in low income groups in 2011[6]; Wang et al. reported the prevalence of overweight among children under 5 was 5.4% in a poor area of mid-western China in 2009[7].

Over the past decade, the child double burden of malnutrition in population level, i.e, the coexistence of under- and overnutrition among children, has received attention and recognition in developing countries[8], and also in urban and rural China[9,10]. But evidence from poor areas, especially among children under 5, are insufficient. Poor areas were often considered to be lack of food resource, and previous studies mostly focused on child protein-energy malnutrition[11]. Meanwhile, child overnutrition, which was mainly due to excessive intake of energy, was expected to be rare in these areas. Therefore, previous policy to improve child nutrition was mainly focused on nutrition supplement [4]. In recent years, the economic conditions have improved in poor areas, and children have access to more food resource than before, so the pattern of child nutrition problems may switch to the double burden of malnutrition[12]. Studies with a representative population of children in poor areas are needed to verify this hypothesis.

United Nations International Children’s Emergency Fund (UNICEF) cooperated with Chinese government have conducted child health project in poor areas of China for several cycles. And the *Multiple indicator cluster survey (MICS) manual* [13] was used to evaluate the nutritional status among children. The previous version of MICS (MICS4) only included indicators of undernutrition, i.e. prevalence of stunting, underweight and wasting. While in 2016, the new version MICS5 added overweight prevalence as an indicator of overnutrition, and used weight for height to evaluate overweight. It was a notable revision, not only for the emphasis of overnutrition but also for the use of weight for height. WHO child growth standards 2006 used to recommend weight for height as the indicator to evaluate wasting, and recommended body mass index(BMI) for age as the indicator of overweight [14]. While in the latest WHO recommendation, the indicator of overweight was revised to weight for height for children under 5 [15]. In previous studies, BMI was widely adopted to evaluate overweight of children under 5 in China [16, 17]. It was necessary to pay attention to the difference of the two evaluation indicators.

A lack of food quality, poor feeding and care practices are often thought to be the primary causes of child malnutrition [18]. Possible risk factors include demographic factors, environmental factors, socioeconomic factors, parental factors, household factors and community factors, etc. However, the results were not consistent among different studies. For example, Jiang et al [19] found caregiver’s education was a risk factor of child stunting, while Wang et al [20] found a null result. Chen et al [21] found children left behind by mothers had higher risk of stunting, but Ban et al [22] found left behind status was not associated with stunting. To ensure the main risk factors may help local government to take effective measures to reduce the prevalence of malnutrition. Additionally, community cluster effect, i.e. the community-level variations should be taken into consideration when conducting analysis.

Among children under 5 in developing countries, malnutrition contributes to more than half of the mortality rate[23]. Malnutrition also has negative effects to children’s physical and mental development [24]. Thus, the aim of this study was to investigate the nutritional status of children under 5 in poor areas of China, especially to evaluate the double burden of malnutrition. Risk factors were also explored to help us understand the child nutritional problems in population level.

## Materials and methods

### Subjects and design

We used data from a cross-sectional study on maternal and child health conducted in 2016, which was funded by UNICEF and National Health and Family Planning Commission of China (NHFPC). The study covered 26 rural counties in 11 provinces of China (Gansu, Qinghai, Xinjiang, Sichuan, Guizhou, Yunnan, Tibet, Henan, Jiangxi, Hebei and Ningxia), and the counties were selected based on their poor socioeconomic development and maternal and child health performance. The average annual income per capita in the survey areas was 5,951 RMB, much lower than the national average (23,821RMB) and rural average (12,363RMB) [25]. Study protocols and procedures were reviewed and approved in accordance with the ethical standards of the Medical Ethics Research Board of Peking University (IRB00001052-16041).

Multistage sampling method was conducted in this study. In China, counties are composed of administrative villages, and one administrative village is composed of several natural villages. Firstly, 15 administrative villages per county and then two natural villages per administrative village were selected randomly with population proportional to size (PPS). Within each selected natural village, 10 households with children under 5 were randomly selected according to the village full registration list of children under 5. In Tibet where the population was sparse, only two or three households with children under 5 were randomly sampled and selected in each natural village. Only the youngest children in each household were selected in the study.

### Data collection

The caregivers of the youngest children in each selected household were face-to-face interviewed using a structured questionnaire, and socioeconomic information (age and gender of child, ethnic groups, education level of the caregiver, left-behind child or not) was collected. We defined the ethnic groups other than the Han group as ethnic minority groups. Left-behind children were defined as mothers left home for work and not live with their children. Written informed consent forms were signed by the caregivers before the interview.

Anthropometric measurement was conducted among children by the investigators. All the investigators were trained using systemic standardized anthropometric training method, and followed the unified anthropometry manuals in the survey. Length was measured for children aged 0–23 months using the SH-8093 Horizontal Type for baby (Suhong Weighing Apparatus Factory, Hengshui, China); height was measured for children aged 24–59 months using the Height Meter with Model SZ-200/120 Type for child (Wujin Weighing Apparatus Factory, Changzhou, China). Weight was measured for all the children using OMRON electronic scale HN-289-BK (OMRON healthcare, Dalian, China). Each measurement was performed twice and the average was taken in the analysis. Length and height were measured to the nearest 0.1cm, and weight was measured to the nearest 0.05kg. Information of data collection was shown in Table 1.

**Table 1:** Data collection of the current study.

	Objects	Contents	Tools
Socioeconomic information	Child caregivers	Age & gender of child, nationality, education level of the caregiver, left-behind child or not	Structured questionnaire
Anthropometric information	The youngest child of each household	Length/Height, weight	Length meter, height meter, weight meter

### Nutritional status evaluation

According to children’s length/height and weight collected in the survey, four evaluation indicators, weight for age, length/height for age, weight for height and body mass index (BMI) for age were used. BMI was calculated using the ratio between children’s weight in kilograms and length/height in meters squared (kg/m^2^). Z score was calculated according to WHO Child Growth Standard 2006[26]. Length/height for age Z score <-2 was defined as stunting; weight for age Z score <-2 was defined as underweight; weight for height Z score <-2 was defined as wasting. Overweight were defined using two indicators. One was that, weight for height Z score >2 defined as overweight; the other was that, BMI for age Z score >2 defined as overweight. Any one or more of the three conditions, stunting, underweight or wasting, was define as undernutrition; overweight evaluating by WHZ was defined as overnutrition. Undernutrition and/or overnutrition was defined as malnutrition.

### Statistical analysis

Each prevalence of malnutrition, i.e., stunting, underweight, wasting, overweight, and the total prevalence of malnutrition were calculated. Categorical variables were compared by Chi-square test. Prevalences evaluated by different indicators among the same sample were compared by McNemar test. A 2-level logistic regression with a second level of community level was used to analysis the possible risk factors of malnutrition. Considering the difference of geographical environments, minority culture, etc. among difference areas, community level was defined as administrative village level in the present study. The modeling was conducted through the following steps: first, model 0 (empty model) examined only within-group homogeneity; second, model 1 included the individual-level explanatory variables. The individual-level variables included month age of children, gender of children, ethnicity, caregiver’s education level, and left-behind children or not. A 2-tailed P value less than 0.05 was considered to be statistically significant. All the analyses were conducted using STATA version 15.0.

## Results

### Descriptive characteristics of participants

A total of 6,570 children and their caregivers were investigated. After excluding 183 children for incomplete core information (e.g. age, anthropometric measurement), and the final sample size for analysis was 6,387. Overall, all the children enrolled were the youngest child in their family and aged 0–59 months. As shown in Table 2, 54.1% were boys and 45.9% were girls. Most of the children were in Han ethnic group (48.3%); the proportion of Tibetan and Yi ethnic group were 11.3% and 10.6%, respectively; while the proportion of other ethnic groups was 29.8%. Most of the caregivers had secondary education, but the proportion of illiteracy was as high as 20.6%, and a quarter of the caregivers had primary education, only 6.2% of the caregivers had college or above education level. The proportion of left-behind children was 15.8%. Overall, the prevalence of malnutrition was 19.2%. The malnutrition rate was statistically different among different age, gender, ethnic groups, caregiver’s education level groups (p<0.05). The prevalence of malnutrition was higher in elder age groups, boys, ethnic minorities (other than Han group) and lower caregiver education level groups.

**Table 2:** Socio-demographic characteristics among children under 5 by malnutrition groups in poor China, 2016.

Characteristics	Total N(%)	Stunting n(%)	Underweight n(%)	Wasting n(%)	Overweight n(%)	Malnutrition n(%)	χ^2^	*p*
Age of child, months								
0–5	605(9.5)	23(4.2)	21(9.4)	43(20.2)	119(21.4)	175(14.3)	59.966	<0.001
6–11	972(15.2)	49(9.0)	46(20.6)	52(24.4)	128(23.0)	213(17.4)		
12–23	1676(26.2)	170(31.4)	61(27.4)	55(25.8)	138(24.8)	342(27.9)		
24–35	1423(22.3)	145(26.8)	52(23.3)	30(14.1)	82(14.7)	241(19.7)		
36–47	930(14.6)	83(15.3)	24(10.8)	20(9.4)	45(8.1)	139(11.3)		
48–59	781(12.2)	72(13.3)	19(8.5)	13(6.1)	45(8.1)	116(9.5)		
Gender of Child								
Boys	3456(54.1)	306(56.5)	120(53.8)	121(56.8)	334(60.0)	715(58.3)	10.829	0.001
Girls	2931(45.9)	236(43.5)	103(46.2)	92(43.2)	223(40.0)	511(41.7)		
Ethnicity								
Han	3088(48.3)	182(33.6)	62(27.8)	84(39.4)	288(51.7)	514(41.9)	53.592	<0.001
Tibetan	719(11.3)	96(17.7)	55(24.7)	41(19.2)	63(11.3)	181(14.8)		
Yi	679(10.6)	110(20.3)	45(20.2)	28(13.1)	47(8.4)	180(14.7)		
Other	1901(29.8)	154(28.4)	61(27.4)	60(28.2)	159(28.5)	351(28.6)		
Caregiver’s education								
Illiteracy	1315(20.6)	180(33.2)	86(38.6)	61(28.6)	93(16.7)	319(26.0)	52.941	<0.001
Primary	1601(25.1)	181(33.4)	72(32.3)	62(29.1)	139(25.0)	354(28.9)		
Secondary	3076(48.2)	169(31.2)	59(26.5)	79(37.1)	289(51.9)	499(40.7)		
College or above	395(6.2)	12(2.2)	6(2.7)	11(5.2)	36(6.5)	54(4.4)		
Left-behind Children								
Yes	1007(15.8)	117(21.6)	40(17.9)	31(14.6)	66(11.8)	197(19.6)	0.104	0.747
No	5380(84.2)	425(78.4)	183(82.1)	182(85.4)	491(88.2)	1029(19.1)		
total	6387(100.0)	542(100.0)	223(100.0)	213(100.0)	557(100.0)	1226(100.0)	-	-

Chi square test was conducted among different groups of malnutrition.

### Double burden of undernutrition and overnutrition

Among the studied population, the prevalence of stunting, was as high as 8.4%; the prevalence of underweight and wasting were 3.5% and 3.3%, respectively (Table 2); overall, the prevalence of undernutrition was 12.0%. On the other hand, the prevalence of overweight was as high as 8.8%, which was even a little higher than the prevalence of stunting. Additionally, 100 children were suffering from both stunting and overweight problems, which accounted for 18.5% (100/542) of all the stunting children and 18.0% (100/557) of all the overweight children.

### Prevalence of overweight evaluated by WHZ and BAZ indicators

As shown in Table 3, the prevalence of overweight among children under 5 evaluated by WHZ and BAZ indicators were 8.8% and 9.9%, respectively. The prevalence of overweight was higher using BAZ indicator than WHZ indicator (p<0.001). In the present study, 77 children would be categorized as normal weight when evaluated by WHZ indicator, but would be re-categorized as overweight when using the BAZ indicator. Further analyses were conducted by different age and gender groups. When compared with the WHZ indicators, in younger age group (0–5 months), BAZ underestimated the prevalence of overweight; and in elder age groups (12–23, 24–35, 36–47 months), the results were overestimated, the differences were statistically significant (p<0.05). In the male group, BAZ overestimated the prevalence of overweight (p<0.001), but in the female group, the difference was not statistically significant (p>0.05).

**Table 3:** Prevalence of overweight and obesity evaluated by WHZ and BAZ indicators.

	WHZ		BAZ		n_2_-n_1_	*p*
	n_1_	%	n_2_	%		
**Age**						
0–5	119	19.7	108	17.8	-11	0.036
6–11	128	13.2	124	12.8	-4	0.302
12–23	138	8.3	189	11.3	51	<0.001
24–35	82	5.8	112	7.9	30	<0.001
36–47	45	4.9	54	5.8	9	0.031
48–59	45	5.8	47	6.0	2	1.000
**gender**						
Boys	334	9.7	387	11.2	53	<0.001
girls	223	7.6	247	8.4	24	0.081
**Total**	557	8.8	634	9.9	77	<0.001

McNemar test was used.

### Risk factors of stunting and overweight

Risk factors of stunting and overweight, i.e. children’s age, gender, ethnicity, caregivers’ education level, left-behind children or not, were studied in multiple logistic regression models. Community effect existed in the model of stunting (ICC = 0.170 in empty model) but not in the model of overweight (ICC = 0.012 in the empty model). Therefore, 2-level logistic regression model was conducted to analysis the risk factors of stunting, and single level logistic regression model was conducted to analysis the risk factors of overweight.

As shown in Table 4, children older than 12 months age, minority, lower caregivers’ education level were found statistically significantly associated with children’s stunting in 2-level multiple logistic regression model (p<0.05).

**Table 4:** Risk factors of stunting among children under 5 in poor China.

		Model 0	Model1		
			OR	95%CI	*P*
Fixed effects					
Age of child, months					
0–5			1	-	
6–11			1.52	0.91,2.56	0.110
12–23			3.39	2.14,5.36	<0.001
24–35			3.56	2.23,5.67	<0.001
36–47			3.27	2.00,5.36	<0.001
48–59			3.60	2.18,5.97	<0.001
Gender of Child					
Boys			1	-	
Girls			0.93	0.77,1.11	0.413
Ethnicity					
Han			1	-	
Tibetan			2.35	1.59,3.48	<0.001
Yi			1.95	1.27,2.99	0.002
Other			1.42	1.07,1.89	0.015
Caregiver’s education					
Illiteracy			3.26	1.74,6.11	0.038
Primary			3.41	1.85,6.28	<0.001
Secondary			1.90	1.04,3.49	<0.001
College and above			1	-	
Left-behind Children					
Yes			1.07	0.83,1.37	0.612
No			1	-	
Random effects					
Variance (SE)		0.667(0.181)	0.388(0.123)		
ICC		0.170	0.105		
Chi-square value		123.08	69.91		
*P*		<0.001	<0.001		

Risk factors of overweight were shown in Table 5. WHZ indicator was used to evaluate child overweight in the regression model, as recommended in the MICS5. Children younger than 24 months age and boys were found statistically significantly associated with overweight in the single level multiple logistic regression model (p<0.05).

**Table 5:** Risk factors of overweight among children under 5 in poor China.

	OR	95%CI	*P*
Age of child, months			
0–5	4.29	2.97,6.19	<0.001
6–11	2.61	1.82,3.72	<0.001
12–23	1.52	1.07,2.16	0.018
24–35	1.02	0.70,1.49	0.908
36–47	0.84	0.55,1.29	0.420
48–59	1		
Gender of Child			
Boys	1		
Girls	0.72	0.60,0.86	<0.001
Ethnicity			
Han	1		
Tibetan	1.14	0.92,1.40	0.229
Yi	1.02	0.73,1.41	0.917
Other	0.70	0.48,1.01	0.058
Caregiver’s education			
Illiteracy	1.00	0.65,1.56	0.985
Primary	1.14	0.77,1.70	0.509
Secondary	1.12	0.78,1.63	0.538
College and above	1		
Left-behind Children			
Yes	0.92	0.69,1.22	0.547
No	1		

## Discussion

In the present study, undernutrition and overnutrition of children under 5 were coexistent in surveyed poor areas of China, and the prevalences of stunting and overweight were obviously high. BAZ tended to overestimate the prevalence of overweight compared with WHZ. Children’s age, gender, ethnicity and caregivers’ education level were associated with children’s stunting and overweight.

### Double burden of malnutrition in poor areas of China

Recent years, the double burden of malnutrition was recognized in some developing countries[8,27,28]. In China, this situation has been reported in some areas with good economic status. For example, Zhang et.al reported the double burden of thinness and overweight among children in Shandong province[9]. Nevertheless, this situation has rarely been paid attention to in poor areas of China, as overweight was generally thought to occur in the urban and economically developed metropolitan areas in China[2]. In the present study, we revealed that the stunting and overweight burdens in current poor China were almost the same high. This result showed that the survey areas was undergoing the nutritional transition, overweight as a new public health problem among children under 5, should be given attention to by the authorities in China even in poor areas.

### Stunting was still a big problem in poor areas of China

Stunting of children under 5 in poor areas of China has been focused for many years. The prevalence of stunting was reported 18.7% in poor rural areas 2013[3]. In the present study, the prevalence of stunting was still high (8.4%), and was found associated with children’s age, ethnic minorities and caregiver’s education level. The prevalence of stunting tended to be higher after 6 months age, and was statistically significant higher after 12 months age, which was probably related to caregivers’ feeding practices. Infants were mainly fed of breast milk during their first 6 months, and introduced to complementary foods after 4–6 months. Previous study reported that children’s caregivers in poor areas of China were lack of feeding information, and cannot gave their children complementary foods in a proper way, including proper starting time, frequency and quality[29]. Lower caregiver’s education was another risk factor against children’s stunting. The result was consistent with previous studies[19]. Caregivers who had higher education levels had better ability to obtain health-related information so that they could give their children balanced diets and nutritious foods, and therefore influence children’s nutritional status[30]. Ethnic minority children, especially the Tibetan and Yi groups, were at high risk of stunting. The result was consistent with previous studies[31], which could possibly explained by traditional culture and feeding habits. Cluster effect of child stunting was also found on administrative village level, which may be probably associated with different geographic living environments (mountain, plateau), and health, economic and education levels among different villages.

### Overweight was a new problem needed to be paid attention

In the present study, WHZ was newly adopted to evaluate overweight among children under 5 according to *MICS5* by UNICEF. While in previous studies, BMI (or BAZ) was widely used in this area[16,32]. According to our result, the prevalence of overweight was lower using WHZ than BAZ, which may avoid overestimate of children’s overweight, and means fewer children will need to control their body weights.

Prevalence of overweight among children under 5 has rarely been reported in poor areas of China. In the present study, the prevalence of overweight was as high as 8.8%, which shown it a newly raising problem. Children’s age and gender were showed associated with overweight. The prevalence of overweight was higher among children under 24 months old and boys. Child overweight was due to excess energy intake, and was more prevalent among children from urban areas and higher income families according to the previous study[6]. While recently, with the economic development, caregivers in poor areas became economically capable to improve children’s feeding. But the lack of correct feeding guidance led to improper feeding practices, e.g., excess energy-intensive foods but less nutrients-intensive foods[16,33]. Boys in rural areas often received more food resources than girls[34], but the improper feeding practices may cause their more energy intake and led to unexpected overweight.

### Recommendations

To solve the problem of child double burden of malnutrition in poor areas, strengthen health education for caregivers should be the first priority. Since short of food resource was no longer the most serious problem in the poor areas we surveyed, interventions should focus on promoting healthy feeding practice and lifestyles to local population. Caregivers should be educated to identify good food resources and scientific feeding practices, and children should be introduce to proper ways of physical exercises.

### Strength

To our knowledge, few studies have focused on the coexistence of under- and over- nutrition among children in poor areas of China, especially children under 5. Cluster effects are not commonly taken into consideration when conducting multivariate analysis. Our study investigated the prevalence of both stunting and overweight of children under 5 in a large population in 11 provinces of China, and studied the socio-economic risk factors using multilevel logistic regression models. The result of our study could contribute to further policies on improving children’s nutritional status and promote child development.

Moreover, WHZ was newly recommended in MICS5 to evaluate overweight of child under 5 from poor areas, and few studies have compared WHZ with BAZ among this specific population. Our study showed the difference of results evaluated by the two indicators and could serve as reference for the following studies.

### Limitation

Some limitations should be considered. First, when sampling, only 2–3 children were selected in each natural village in Tibet, where is a vast territory with a sparse population. Though it cost large amount of manpower and material resources to survey there, we could hardly interview more households. The small sample size in Tibet may not well represent the local condition. Larger survey was still needed in the future in Tibet. Second, the study was a cross-sectional design, which cannot explain the causal relationships between child malnutrition and the risk factors. Further prospective study design was needed. Moreover, factors in community level were not included, and only a few individual level factors have been considered in the present study. Family income was failed to be included in the logistic analysis. One reason was that most caregivers we interviewed were females, and some of them did not know the economic status of their family. Another reason was that some respondents treated the family income as private information and did not want to report. Feeding practice detail information was not included in the present study, either. In the further study, more relative factors should be considered.

In conclusion, our study showed the coexistence of under and over nutrition among children under 5 in poor areas of China. BAZ overestimated the prevalence of overweight compared with WHZ. Children over 6 months old, lower caregivers’ education level and ethnic minorities were the risk factors of stunting; children under 24 months old and gender of boys were the risk factors of overweight. Health education should be provided to caregivers in these areas to improve children’s health.
